# Characterization of isoprene-derived secondary organic aerosols at a rural site in North China Plain with implications for anthropogenic pollution effects

**DOI:** 10.1038/s41598-017-18983-7

**Published:** 2018-01-11

**Authors:** Jianjun Li, Gehui Wang, Can Wu, Cong Cao, Yanqin Ren, Jiayuan Wang, Jin Li, Junji Cao, Limin Zeng, Tong Zhu

**Affiliations:** 1 State Key Laboratory of Loess and Quaternary Geology, Key Laboratory of Aerosol Chemistry and Physics, Institute of Earth Environment, Chinese Academy of Sciences, Xi’an, China; 2 Key Laboratory of Geoscience Information of the Ministry of Education, School of Geographic Sciences, East China Normal University, Shanghai, China; 3 Center for Excellence in Regional Atmospheric Environment, Institute of Urban Environment, Chinese Academy of Sciences, Xiamen, China; 4 University of Chinese Academy of Sciences, Beijing, China; 5 BIC-ESAT and SKL-ESPC, College of Environmental Sciences and Engineering, Peking University, Beijing, China

## Abstract

Isoprene is the most abundant non-methane volatile organic compound (VOC) and the largest contributor to secondary organic aerosol (SOA) burden on a global scale. In order to examine the influence of high concentrations of anthropogenic pollutants on isoprene-derived SOA (SOA_*i*_) formation, summertime PM_2.5_ filter samples were collected with a three-hour sampling interval at a rural site in the North China Plain (NCP), and determined for SOA_*i*_ tracers and other chemical species. RO_2_+NO pathway derived 2-methylglyceric acid presented a relatively higher contribution to the SOA_*i*_ due to the high-NOx (~20 ppb) conditions in the NCP that suppressed the reactive uptake of RO_2_+HO_2_ reaction derived isoprene epoxydiols. Compared to particle acidity and water content, sulfate plays a dominant role in the heterogeneous formation process of SOA_*i*_. Diurnal variation and correlation of 2-methyltetrols with ozone suggested an important effect of isoprene ozonolysis on SOA_*i*_ formation. SOA_*i*_ increased linearly with levoglucosan during June 10–18, which can be attributed to an increasing emission of isoprene caused by the field burning of wheat straw and a favorable aqueous SOA formation during the aging process of the biomass burning plume. Our results suggested that isoprene oxidation is highly influenced by intensive anthropogenic activities in the NCP.

## Introduction

Atmospheric secondary organic aerosols (SOA) are produced from photochemical oxidation products of volatile organic compounds (VOCs) via gas-particle conversion processes such as nucleation, condensation and heterogeneous chemical reactions^1^. SOA account for about 70 percent of global organic aerosols in the troposphere^1^, and have important impacts on climate and human health^2–5^. Among all the precursors isoprene is the most important contributor to the global SOA burden^6,7^. Annual isoprene emission on the Earth surface is about 600 Tg yr^−1^ ^8^, comprising approximately half of the total VOC emissions from both natural and anthropogenic sources^7,9^. On a global scale, isoprene-derived products is about 19.2 TgC yr^−1^, accounting for ~70% of the total SOA^10^.

A number of studies found that oxidation of isoprene by hydroxyl radical (OH) generates large amounts of aerosol phase low-volatility products^6,11–13^. Under low-NO_x_ conditions isoprene reacts with OH and HO_2_ radicals and produces isoprene epoxydiols (IEPOX)^12,14,15^, while under high-NOx conditions isoprene reacts with OH radical and NOx, producing methacrylic acid epoxide (MAE)^16^ and hydroxymethyl-methyl-lactone (HMML)^17^. Those epoxides are critical intermediates of isoprene SOA formation through reactive uptake by acidic particles. In addition, recent studies found that ozonolysis of isoprene leads to stabilized Criegee intermediates (sCIs), which could also be an important contributor to isoprene-derived SOA (SOA_*i*_) in the atmosphere^18,19^. Moreover, many studies have found that relative humidity (RH)^20,21^, aerosol acidity^22–24^, anthropogenic pollutants such as gaseous NO_x_ and SO_2_^25–27^ and particulate sulfate^13,14^ have pronounced effects on SOA_*i*_ formation.

In comparison with other countries, the atmospheric environment of China has been suffering from high levels of SO_2_, NO_x_, O_3_, VOCs, NH_3_ and particulate matter (PM) especially in North China Plain (NCP). Due to the lack of efficient emission controls during the fast industrialization and urbanization processes, severe haze episodes frequently occurred in the region in the past decades^28,29^. In addition, field open burning is still a common activity for disposal of crop residue in the rural area of NCP, especially in summertime wheat harvest period, which releases huge amount of pollutants into the atmosphere and leads to significant impacts on the air quality and aerosol properties^30–32^.

Many previous studies in the NCP focused on anthropogenic source distributions of gaseous and particle pollutants, and their environmental, ecological and human health effects^33–36^. However, only few studies examined how biogenic SOA is affected by such high loadings of anthropogenic pollutants in the region^37–39^. In the current study, PM_2.5_ filter samples were collected with a three-hour sampling interval at a rural site in the central part of NCP during June 10^th^ to 25^th^, 2013, and determined for SOA_*i*_ tracers to explore the influence of anthropogenic pollution on SOA_*i*_ formation. We firstly investigated the temporal variation and chemical composition of SOA_*i*_ in NCP_,_ and then examined the impacts of sulfate, ozone and biomass burning on the SOA_*i*_ formation.

## Results and Discussion

### Temporal variation of isoprene-derived SOA tracers

**Figure 1:**
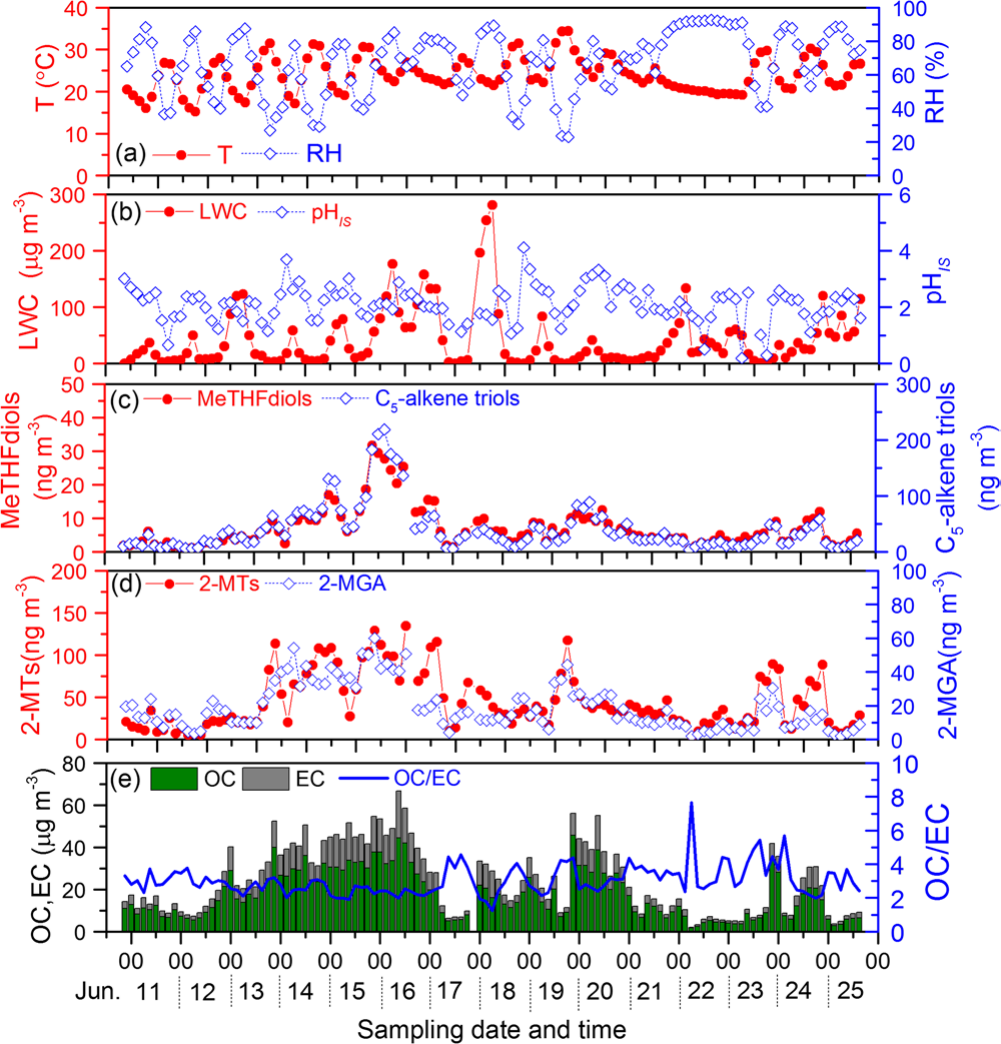
Temporal variations of biogenic secondary organic aerosols (BSOA) derived from isoprene, temperature, relative humidity, pH_IS_ (represents particle acidity), LWC (liquid water content), OC and EC (organic and elemental carbon). MeTHFdiols: the sum of cis-3-methyltetrahydrofuran-3,4-diol and trans-3-methyltetrahydrofuran-3,4-diol; C_5_-alkene triols: the sum of 3-methyl-2,3,4-trihydroxy-1-butene, cis-2-methyl-1,3,4-trihydroxy-1-butene, and trans-2-methyl-1,3,4-trihydroxy-1-butene; 2-MTs: the sum of 2-methylthreitol and 2-methylerythritol; 2-MGA: 2-methylglyceric acid.

3-Methyltetrahydrofuran-3,4-diols (3-MeTHF-3,4-diols), 2-methylglyceric acid, C_5_-alkene triols and 2-methyltetrols were detected in all the filter samples collected at the Gucheng Meteorological Station (Table 1). Among the eight isoprene-derived SOA tracers, 3-MeTHF-3,4-diols, C_5_-alkene triols and 2-methyltetrols are mainly formed by reactive uptake of IEPOX, key intermediates that are produced by RO_2_+HO_2_ reactions of isoprene without involvement of NO_x_^12,14^. The gas-phase IEPOX can be trapped into the aerosol phase through acid-catalyzed intermolecular rearrangement reactions to form 3-MeTHF-3,4-diols and/or C_5_-alkene triols^40,41^. Thus, 3-MeTHF-3,4-diols and C_5_-alkene triols at Gucheng presented a similar temporal variation trend (Fig. 1c) with a strong linear correlation (*r*^2^ = 0.91, Figure S2a). 2-Methyltetrols are primarily formed through the nucleophilic addition of water to the ring-opened products of the epoxydiols in aerosol phase^12^, which is a more complex pathway compared to 3-MeTHF-3,4-diols and C_5_-alkene triols formation. Moreover, Riva *et al*.^18^ recently demonstrated that isoprene ozonolysis could be another source for 2-methyltetrols in the presence of acidic sulfate aerosol (detailed mechanism discussed in the following section). Thus it is reasonable that correlation of 2-methyltetrols with 3-MeTHF-3,4-diols (*r*^2^ = 0.66) or C_5_-alkene triols (*r*^2^ = 0.54) (Figs 1 and S2b) was not as strong as 3-MeTHF-3,4-diols and C_5_-alkene triols.

**Table 1:** Concentration (ng m^−3^) of isoprene-derived SOA tracers in the summertime PM_2.5_ of Gucheng, Hebei province in China.

SOA tracers	Daytime				Night time			
	Min	Max.	Mean	SD	Min.	Max.	Mean	SD
(I) 3-MeTHF-3,4-diols								
*trans*-3-methyltetrahydrofuran-3,4-diol	0.56	10.6	2.6	2.0	0.27	12.9	3.2	2.8
* cis*-3-methyltetrahydrofuran-3,4-diol	0.63	14.8	3.6	2.7	0.46	18.9	4.7	4.1
(II) C_5_-alkene triols								
*cis*-2-methyl-1,3,4-trihydroxy-1-butene	1.2	41.5	8.0	7.4	1.1	54.5	11.4	12.0
3-methyl-2,3,4-trihydroxy-1-butene	2.3	49.7	11.8	9.7	1.6	61.3	15.6	14.4
*trans*-2-methyl-1,3,4-trihydroxy-1-butene	2.2	73.7	14.0	13.2	1.6	104.9	20.5	22.9
(III) 2-MTs (2-methyltetrols)								
2-methylthreitol	2.1	51.0	16.4	11.7	1.2	48.9	17.9	14.3
2-methylerythritol	3.8	83.6	27.6	18.6	2.8	80.4	28.1	20.9
(IV) 2-MGA								
2-methylglyceric acid	3.2	51.0	19.3	13.1	2.0	60.0	19.0	13.8

Under high-NOx conditions isoprene can be oxidized into methacrolein (MACR) through RO_2_+NO reaction, which undergoes further oxidation with OH and NOx radicals to form methacryloyl peroxynitrate (MPAN)^25,42^. MPAN may react with OH radical to produce MAE and HMML. Those two compounds undergo further reactions under acid conditions, forming 2-methylglyceric acid and some related dimers, organic nitrates and organosulfates^17,27^. Field studies conducted at low-NOx sites (0.1–2 ppb) in the US^27,43,44^ and Germany^45^ showed that 2-methylglyceric acid concentrations are almost one order of magnitude lower than 2-methyltetrols and C_5_-alkene triols. In contrast, NOx levels (around 20 ppb)^33^ in NCP are much higher. Thus, the role of RO_2_+NO pathway in SOA_*i*_ formation is more important in the region. As shown in Table 1, concentrations of 2-methylglyceric acid (2.0–60 ng m^3^, ave.19 ng m^3^) in the PM_2.5_ samples at the Gucheng site are only 2–4 times lower than those of 2-methyltetrols (4.0–134 ng m^3^, ave. 45 ng m^3^) and C_5_-alkene triols (4.3–219 ng m^3^, ave. 41 ng m^3^). Coincidentally, a recent real-time observation in Eastern China (Nanjing) revealed a suppression of IEPOX-SOA formation caused by the low IEPOX reactive uptake rate and NO-dominant gas-phase chemistry under high-NOx conditions (21.4 ppb)^46^. Both results highlight the important role of the severe anthropogenic pollution in isoprene-derived SOA formation process in the NCP atmosphere.

All the SOA_*i*_ tracers showed a similar temporal variation pattern to organic carbon (OC) (Fig. 1e); concentration of total SOA_*i*_ tracers linearly correlated with that of OC with a coefficient of *r*^2^ = 0.61, suggesting that isoprene oxidation is an important source of organic aerosols in the rural area of NCP.

### Influence of sulfate, aerosol acidity and water content

Acid-catalyzed reactive uptake and subsequent particle-phase reactions of IEPOX (for 3-MeTHF-3,4-diols, C_5_-alkene triols and 2-MTs) and MAE or HMML (for 2-MGA) are the most important chemical pathways of SOA formation from isoprene^12,16^. Several previous studies found that aerosol particles of varying acidity and liquid water contents can affect the SOA_*i*_ yield^22,23,47,48^. In order to examine the aerosol aqueous chemistry during the SOA_*i*_ formation, ISORROPIA-II, a thermodynamic model, was performed to estimate the aerosol acidity (i.e., *In-situ* pH, pH_*IS*_, or H^+^ concentration in the aqueous phase, H^+^_aq_) and liquid water content (LWC). As mentioned in Methods section, pH values could approximately be underestimated by one unit due to the lack of NH_3_ data^49,50^. In the current study, pH values of PM_2.5_ ranged from 0.2 to 4.1 with an average of 2.1 ± 0.6 at the Gucheng site (Fig. 1b), indicating that the acidity of aerosol in the rural region of NCP is much weaker than that in southeast US (0.5–2.0)^49^, mainly due to the abundant alkaline NH_3_ in the region^29^. In contrast, aerosol water content (42 ± 50 μg m^−3^) at the Gucheng site is about 10 times higher than that (5.1–8.4 μg m^−3^) in southeastern US^48^, which can be ascribed to the high loading of inorganic ions in NCP.

**Figure 2:**
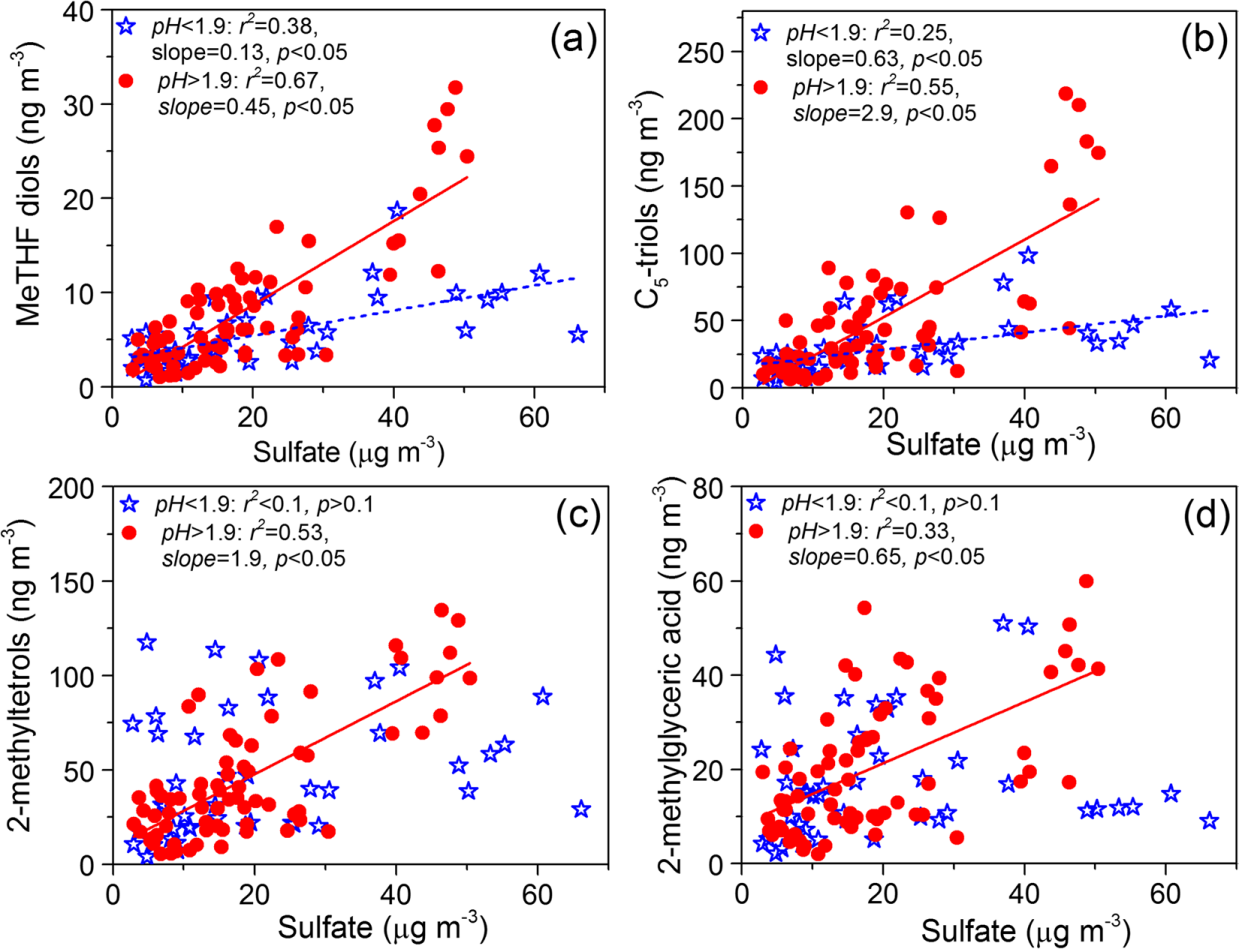
Relationships between sulfate and determined isoprene-derived products.

Based on a multivariate linear regression analysis of SOA_*i*_ tracers (*r*^2^ = 0.41, *p* < 0.001) with sulfate, particle acidity (represented as H^+^_aq_), and water content, we found that only sulfate has a statistically significant (*p* < 0.001) positive linear relationship with SOA_*i*_ tracers (Table S1). Moreover, Fig. 2 showed that all the detected SOA_*i*_ tracers present moderate or good linear correlation (*r*^2^ = 0.3~0.6) with sulfate concentration, again demonstrating the enhancing effect of sulfate on SOA_*i*_ formation, which is consistent with recent studies in the southeast US^43,44,48,51^. Such an effect can be primarily explained by two reasons: 1) acidic sulfate provides a surface that is favorable for acid-catalyzed reactive uptake and ring-opening reaction of the key intermediates in the gas phase^14,43^, 2) salting-in effect of sulfate increases the solubility of polar organic compounds like IEPOX, MAE and HMML^48^. However, as shown in Fig. 2, we found that sulfate and SOA_*i*_ tracers present stronger correlations and higher slopes in lower acidity conditions (pH > 2 in the current study, the red dots in Fig. 2), which indicates that reactive acidic uptake of SOA_*i*_ precursors is more responsible for sulfate growth in the atmosphere when aerosols acidity is weak. This phenomenon may be related to the effects of sulfate on IEPOX reaction probability (γ_IEPOX_), aerosol acidity^52,53^, surface area^54^, and the exact explanation needs further research.

In agreement with the multivariate regression analysis, no significant correlations of the detected SOA_*i*_ tracers with particle acidity and liquid water content (LWC) of aerosol were found in the current study (Fig. 1b), consisting with the results observed in southeastern US^43,48^. This is because that the influences of aerosol acidity and water content on isoprene oxidation are very complicated processes in ambient environment, which can also involve in viscosity or morphology changes and liquid–liquid phase separations of aerosols, dilution effects on ion strength and acidity by particle-phase water, and competitions from gas phase oxidation^48,55^. In addition, particle acidity and liquid water content may vary regionally, which can also influence their relationships to SOA_*i*_ concentration in the sampling site when SOA are formed during a long-distance transport process.

### Influence of ozone

**Figure 3:**
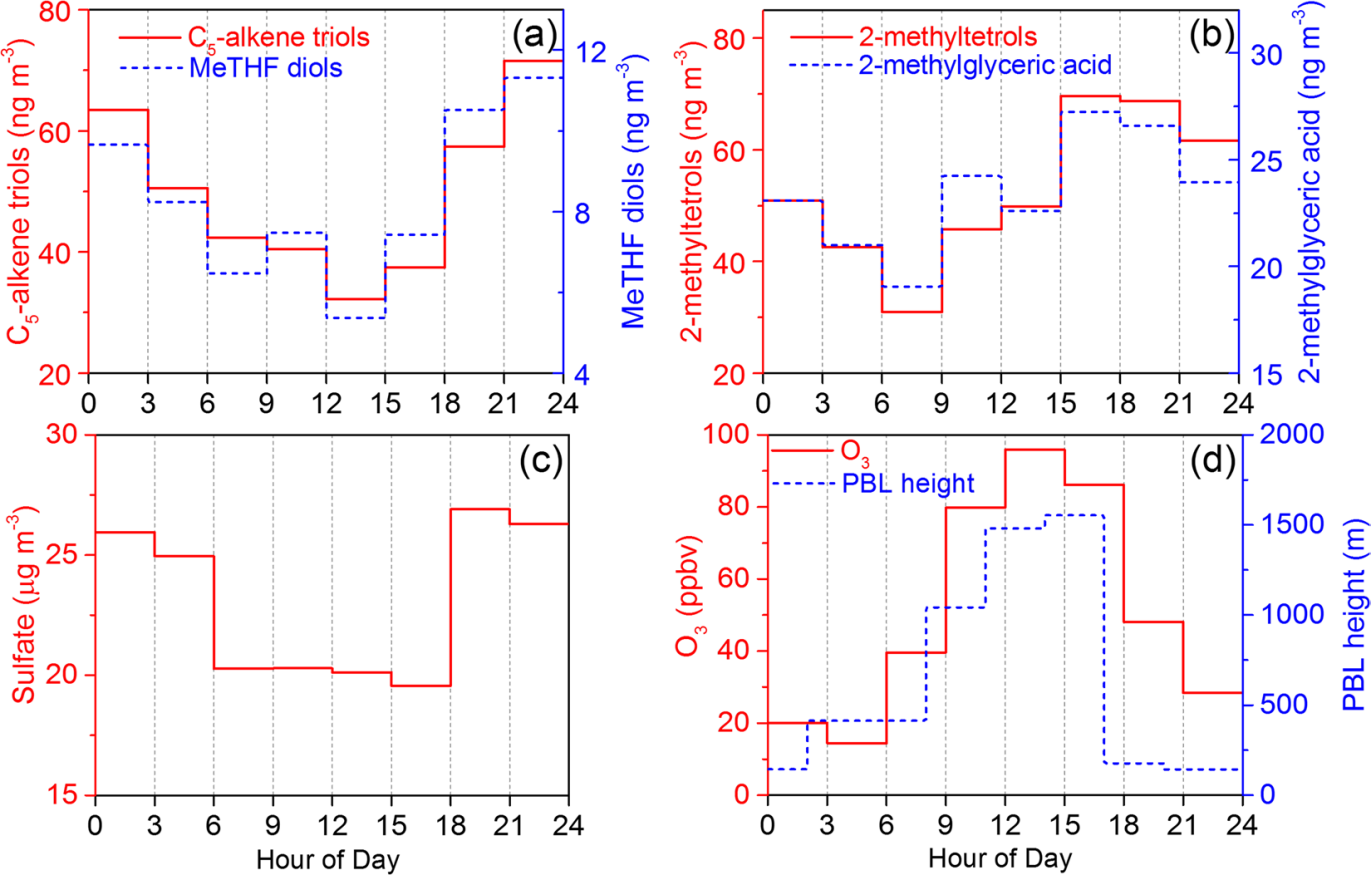
Diurnal variation of the determined isoprene-derived products, sulfate, ozone and Planetary Boundary Layer (PBL) height (data provided by Real-time Environmental Applications and Display System, https://ready.arl.noaa.gov/READYamet.php) during June 11–19.

Figure 3 displays diurnal variations of the SOA_*i*_ tracers at Gucheng station during June 11–19, 2013, which was a period without precipitation. Both 3-MeTHF-3,4-diols and C_5_-alkene triols presented higher concentrations in the nighttime than in the daytime (Fig. 3a and b) with a statistic significance (*p* < *0*.*05*). The planetary boundary layer (PBL) height decrease (Fig. 3d) due to the nighttime lower temperature (Fig. 1) would be the primary reason. On the other hand, as showed in Fig. 3c, higher concentration of sulfate can enhance the yield of isoprene-derived SOA by promoting gas-aerosol phase conversion of its precursors and the subsequent aqueous phase chemistry. Thus, the diurnal variation of both 3-MeTHF-3,4-diols and C_5_-alkene triols showed a maximum at 21:00–24:00 p.m. and a minimum in the afternoon (12:00–15:00 p.m.).

**Figure 4:**
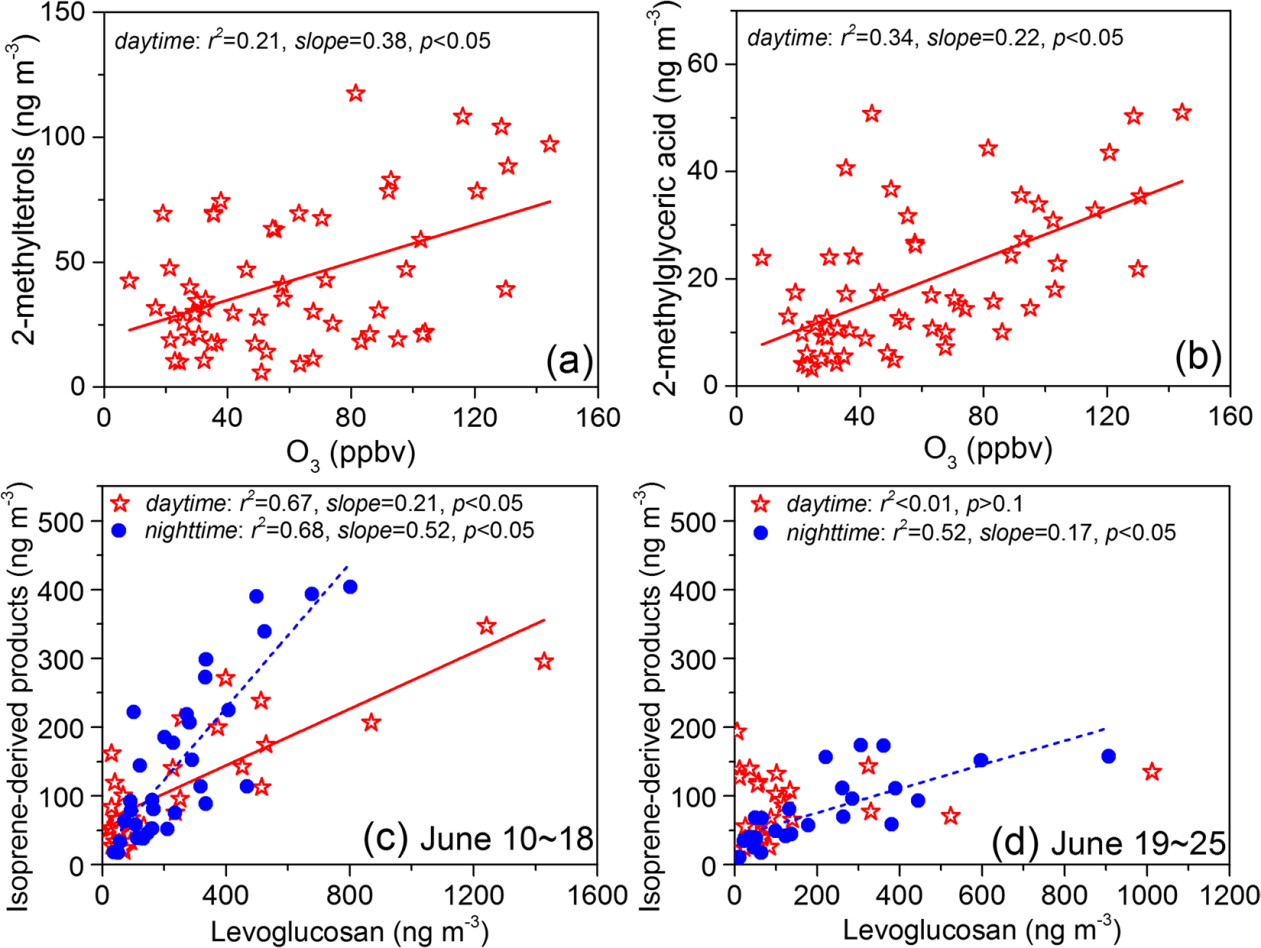
Relationships of (**a**) 2-methylthetrols and (**b**) 2-methylglyceric acid with ozone during the whole sampling period, and total determined isoprene-derived products with levoglucosan during (**c**) June 10~18 and (**d**) June 19–25.

However, 2-methyltetrols showed a diurnal variation pattern different from that of 3-MeTHF-3,4-diols and C_5_-alkene triols. Concentrations of 2-methyltetrols displayed an obvious increase in the afternoon, peaking at 15:00–18:00 p.m. In addition to •OH-initiated oxidation, isoprene ozonolysis is also a potential pathway of SOA formation^56^. Previous studies^19,57^ proposed that initial formation of isoprene primary ozonides leads to stabilized sCIs, which can further react in the gas phase to form higher molecular weight products that subsequently partition to the aerosol phase and form SOA. Based on their chamber study, Riva *et al*.^18^ found that in the presence of wet acidic aerosols isoprene ozonolysis yields 2-methyltetrols and organosulfates but not produces C_5_-alkene triols, 3-MeTHF-3,4-diols and 2-methylglyceric acid. They tentatively proposed that hydroperoxides formed in the gas phase from isoprene ozonolysis potentially partition to wet acidic sulfate aerosols and hydrolyze to 2-methyltetrols and related organosulfates. Recently, Rattanavaraha *et al*.^44^ verified that isoprene ozonolysis plays a role in 2-methyltetrols formation process. During the current study period, ozone showed a highest concentration in the afternoon (12:00–15:00 p.m.) (Fig. 3d), and weakly correlated with 2-methyltetrols (*r*^2^ = 0.21, Fig. 4a) in the daytime, suggesting an important role of isoprene ozonolysis in the SOA formation process in NCP. Additionally, the non-correlation of ozone with 3-MeTHF-3,4-diols and C_5_-alkene triols and the different diurnal variation patterns of those SOA_*i*_ tracers suggest that isoprene ozonolysis would have limited influence on these compounds.

In consistent with the field study by Rattanavaraha *et al*.^44^, 2-methylglyceric acid, represented as MAE/HMML-derived SOA, also presented a moderate correlation (r^2^ = 0.34, Fig. 4b) with O_3_ concentration during the daytime, and was highest during 15:00–18:00 p.m., because the formation of MACR (a precursor to MAE and HMML) was enhanced by oxidation of isoprene by O_3_^44^.

### Influence of regional biomass burning

**Figure 5:**
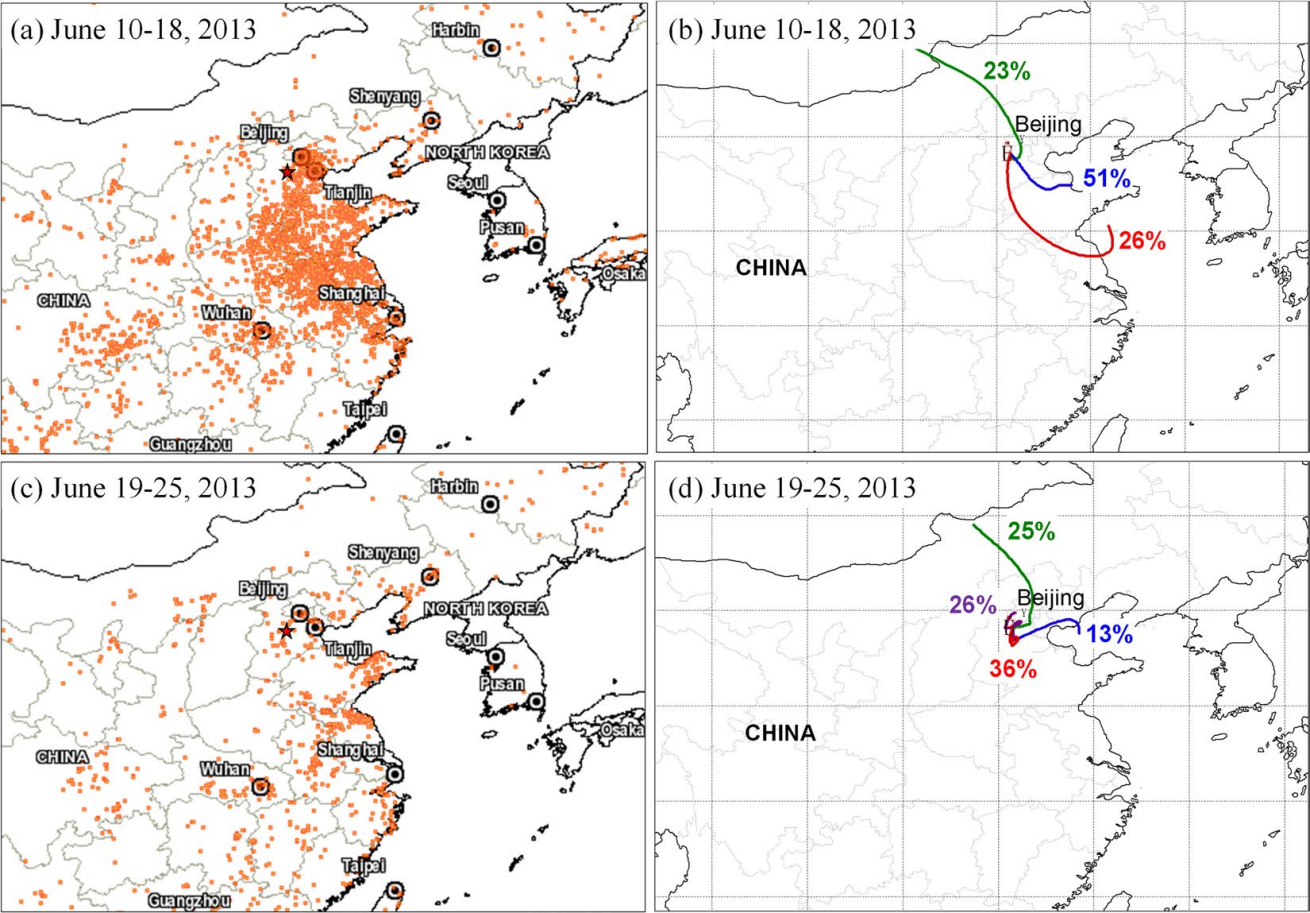
Fire spots (**a**,**c**) (provided by Fire Information for Resource Management System, FIRMS, https://firms.modaps.eosdis.nasa.gov/firemap/, Collection 6 and MCD14ML), and cluster analyses of air masses (**b**,**d**) (original data of backward trajectories provided by NOAA HYSPLIT modeling system^68,69^, http://ready.arl.noaa.gov/HYSPLIT.php) during the sampling period (June 10–18 and June 19–25, 2013). Sampling site represented as red star.

From the end of May to the mid of June is the wheat harvest season and field open burning of wheat straw is a common activity in the rural area of NCP. Based on the NASA satellite observation of fire spots (https://firms.modaps.eosdis.nasa.gov/firemap/) (Fig. 5a), we found that intensive emissions from the wheat straw burning occurred during the first half of the sampling period (i.e., June 10–18, 2013) in the NCP region. As shown in Fig. 5b, 77% of the air masses arriving at the Gucheng station during June 10–18 were transported via long distances from the southeast part of NCP. Some emission inventory studies revealed that biomass burning could release large amounts of isoprene to the atmosphere^58,59^. Zhu *et al*.^60^ examined the amounts of VOCs in the air at another rural site (Yucheng, Shandong Province, China) in the NCP region, and found that isoprene concentration during the wheat straw burning period is around double of that in normal periods. Thus, in order to discuss the influence of the regional intense burning of biomass fuels on isoprene-derived SOA in the rural areas of NCP, the relationships between SOA_*i*_ tracers and biomass burning tracer (i.e., levoglucosan) were analyzed (Fig. 4c and d). During June 10–18, the detected SOA_*i*_ tracers positively correlated with levoglucosan with a high coefficient (*r*^2^ = 0.67 in the daytime and 0.68 in the nighttime) (Fig. 4c), suggesting that emissions from the biomass burning enhanced the isoprene-derived SOA formation in the NCP region.

As shown in Fig. 5c and d, during the late half of sampling period (i.e., June 19–25), the number of fire spots decreased sharply, suggesting that field burning biomass during the period was insignificant. Thus the correlation between the SOA_*i*_ tracers and levoglucosan during the period was not as significant as that during June 10–18 (Fig. 4d). However, the SOA_*i*_ tracers and levoglucosan still had a moderate relationship (*r*^2^ = 0.52) in the nighttime during June 19–25. As shown in Fig. 5d, most air masses (62%) of Gucheng during the late half period were transported from the local area. Because at high isoprene emission from natural plants is insignificant and the local residential biomass burning is the major source, thus the correlation of the SOA_*i*_ tracers with levoglucosan was only observed at night. Interestingly, the nighttime SOA_*i*_ tracer concentrations during June 10–18 (150 ± 115 ng m^−3^) were obviously higher than those in the period of June 19–25 (85 ± 53 ng m^−3^) (Table S2), although the nighttime concentrations of levoglucosan during the two phases (250 ± 182 and 218 ± 208 ng m^−3^) were comparable. Recently, Gilardoni *et al*.^61^ directly observed a strong formation of aqueous secondary organic aerosol from biomass burning emissions. At the Gucheng site nighttime concentration (26 ± 16 μg m^−3^) of sulfate during June 10–18 was more than 2 times higher than that (12 ± 11 μg m^−3^) in the remaining period (Table S2), which can be attributed to a continuous aqueous oxidation of SO_2_ from biomass burning and other anthropogenic activities during the long-range transport at the first period. As discussed in Section 3.2, sulfate plays an important role in promoting aqueous phase oxidation of isoprene-derived products. Thus, the higher SOA_*i*_ concentration during the nighttime of June 10–18 was attributed to an enhancing aqueous-phase chemistry of isoprene-derived organics during the biomass burning smoke transport process.

## Methods

### Field Sampling

The measurement was performed at the Integrated Ecological-Meteorological Observation and Experiment Station of Chinese Academy of Meteorological Sciences (39°08′N, 115°40′E, 15.2 m asl, Figure S1), which is located in a rural area of Gucheng, Hebei Province. Detailed information of the station and some observation results of meteorological condition and air quality in the area were described elsewhere^33^. Briefly, the Gucheng site is in the central zone of Beijing-Tianjin-Hebei Region, about 110 km southwest to Beijing (See Figure S1). Taihang Mountains, with a range extending over 400 km from north to south, is about 30 km away from the west of the site. The sampling station is surrounded by the farms and villages, where the main crops are corn and wheat in summer. Weather conditions during the observation period are characteristic of warm and humid (24 ± 4 °C and 67 ± 19%). In all the seasons, the prevailing surface wind directions were northeasterly and southwesterly. A previous study showed that the concentrations of NO_x_, SO_2_, and O_3_ in June (2007) were around 20 ppb, 10 ppb, and 50 ppb, respectively, indicating severe air pollution in summer in the region^33^.

PM_2.5_ samples were collected on the rooftop (about 10 m above the ground) of a three-story building at the site of the Gucheng station. The sampling was conducted from June 10^th^ to 25^th^, 2013 by using a high volume (1.13 m^3^ min^−1^) sampler (Anderson) with a three-hour sampling interval. A total of 118 field samples were collected onto pre-baked (450 °C, 6–8 hr) quartz fiber filters (20.3 cm * 25.4 cm). Three field blank samples were also collected by mounting blank filters onto the sampler for about 15 min without pumping any air. After sampling, the sample filter was individually sealed in pre-baked aluminum foil bags and stored in a freezer (−20 °C) prior to analysis.

### Chemical analysis

A punch of the filter (including both field and blank samples, N = 121) with an area of 12.5–25 cm^2^ was extracted three times with a mixture of dichloromethane and methanol (2:1, v/v) each for 10 min under ultrasonication. The extracts were concentrated using a rotary evaporator under vacuum conditions and then blow down to dryness using pure nitrogen. After reaction with 50 μL N,O-bis-(trimethylsilyl) trifluoroacetamide (BSTFA) with 1% of trimethylsilyl chloride and 10 μL of pyridine at 70 °C for 3 hr, the derivatives were determined using gas chromatography/electron ionization mass spectrometry (GC/EI-MS)^55^.

GC/EI-MS analysis of the derivatives was performed using an Agilent 7890 A GC coupled with an Agilent 5975 C MSD. GC oven temperature program and MS conditions were described elsewhere^55,62,63^. Individual characteristic ions, m/z 262 for 3-MeTHF-3,4-diols (trans-3-methyltetrahydrofuran-3,4-diol and cis-3-methyltetrahydrofuran-3,4-diol), m/z 219 for 2-MGA (2-methylglyceric acid), m/z 231 for C_5_-alkene triols (cis-2-methyl-1,3,4-trihydoxy-1-butene, 3-methyl-2,3,4-trihydoxy-1-butene and trans-2-methyl-1,3,4-trihydoxy-1-butene), and m/z 219 for 2-MTs (2-methylthreitol and 2-methylerythritol), are used for quantification of the isoprene-derived SOA tracers. However, the response factors of all the determined products are replaced by erythritol, because the authentic standards are not commercially available^27,37^. No significant contamination (<5% of those in the samples) was found in the blanks. In addition, it is worth nothing that recent studies have argued that part of detected SOA_i_ tracers like 3-MeTHF-3,4-diols, C_5_-alkene triols and 2-methyltetrols are decomposition products of low-volatile IEPOX accretion products (like oligomers of IEPOX), which could occur during thermal desorption and/or derivatization heating processes^64,65^. Thus the tracers in the current study were possibly somewhat overestimated.

OC and EC in the PM_2.5_ samples were analyzed using DRI Model 2001 Carbon analyzer following the Interagency Monitoring of Protected Visual Environments (IMPROVE) thermal/optical reflectance (TOR) protocol. Water soluble inorganic ions including SO_4_^2−^, NO_3_^−^, NH_4_^+^, Cl^−^, Ca^2+^, K^+^, and Mg^2+^ in samples were analyzed using ion chromatography. Detailed methods for OC, EC, and ions determination are provided in the supplementary material.

### Estimations of aerosol pH and water content

ISORROPIA-II^66,67^, a thermodynamic model, was used to estimate aerosol pH and water content, because a direct measurement of aerosol acidity (i.e., pH) is not possible at present. Following by the assumptions of Weber *et al*.^49^, the PM_2.5_ aerosol at the Gucheng rural site was considered as internally mixed and in a single aqueous phase that contained the inorganic species, and no compositional dependence on particle size. In applying ISORROPIA-II, “forward” mode calculation, in which inputs to the model include temperature (T), relative humidity (RH), and the total (gas+aerosol) concentrations of aerosol precursors in the air parcel, would be more suitable to the aerosol pH and water content estimation, although the concentration of NH_3_, HNO_3_, and HCl in the gas phase are unavailable in the current study^50^. Therefore, the pH results given by the current work would be systematically underestimated by approximately one unit^49,50^.

## Electronic supplementary material

Supplementary Information
