# Supplementary material for: Characterization of isoprene-derived secondary organic aerosols at a rural site in North China Plain with implications for anthropogenic pollution effects
Source: Sci Rep. 2018 Jan 11;8:535. doi: 10.1038/s41598-017-18983-7 (PMC5765163; doi:10.1038/s41598-017-18983-7)
Supplement: Supplementary file 1 — Supplementary Information [file 41598_2017_18983_MOESM1_ESM.pdf]

Supporting information

Characterization of isoprene-derived secondary organic aerosols at a rural site in North China Plain with implications for anthropogenic pollution effects

Jianjun Li[1], Gehui Wang[1,2,3,4,*], Can Wu[1,4], Cong Cao[1,4], Yanqin Ren[1,4], Jiayuan Wang[1,4], Jin Li[1.4], Junji Cao[1], Limin Zeng[5], Tong Zhu[5,*]

[1] State Key Laboratory of Loess and Quaternary Geology, Key Laboratory of Aerosol Chemistry and Physics, Institute of Earth Environment, Chinese Academy of Sciences, Xi'an, China

[2] Key Laboratory of Geoscience Information of the Ministry of Education, School of Geographic Sciences, East China Normal University, Shanghai, China

[3] Center for Excellence in Regional Atmospheric Environment, Institute of Urban Environment, Chinese Academy of Sciences, Xiamen, China

[4] University of Chinese Academy of Sciences, Beijing, China

[5] BIC-ESAT and SKL-ESPC, College of Environmental Sciences and Engineering, Peking University, Beijing, China

*Corresponding authors:
Prof. Gehui Wang, E-mail: wanggh@ieecas.cn or ghwang@geo.ecnu.edu.cn;
Prof. Tong Zhu, E-mail: tzhu@pku.edu.cn

Measurements of elemental carbon (EC) and organic carbon (OC)

OC and EC in the $PM_{2.5}$ samples were analyzed using DRI Model 2001 Carbon analyzer following the Interagency Monitoring of Protected Visual Environments (IMPROVE) thermal/optical reflectance (TOR) protocol (Chow et al., 2004, 2007). A size of 0.526 cm^2 sample filter was put in a quartz boat inside the analyzer and stepwise heated to temperatures of 140℃ (OC1), 280℃ (OC2), 480℃ (OC3), and 580℃ (OC4) in a non-oxidizing helium (He) atmosphere, and 580℃ (EC1), 740℃ (EC2), and 840℃ (EC3) in an oxidizing atmosphere of 2% oxygen in helium. In addition, pyrolized carbon (PC) is determined by reflectance and transmittance of 633 nm light. The analyzer was calibrated with known quantities of CH_4 every day. One of 10 samples was analyzed in replicates. Three blank filters were analyzed and subtracted from the ambient measurements. Differences determined from replicate analyses were <5% for TC, and <10% for OC and EC for this study.

Determination of water soluble inorganic ions

A size of 12.5-25 cm^2 punch aliquot from $PM_{2.5}$ sample was extracted with 50 mL pure water respectively and filtered through a PTFE filter to remove the particles and filter debris. Then the water-extracts were determined for inorganic ions using an ion chromatography (Dionex 500, Dionex, US). Five cations (Na^+, K^+, NH_3^+, Mg^{2+} and Ca^{2+}) and six anions (F^-, Cl^-, Br^-, NO_2^-, NO_3^-, and SO_4^{2-}) were determined. The limits of detection were less than 0.05 mg L^{-1} for anions and cations. Br^- and NO_2^- were not discussed in this paper because their mass concentrations were below the detection limit. Standard Reference Materials produced by the National Research Center for

Certified Reference Materials, China, were analyzed for quality assurance purposes. All data reported here were subtracted by the field blanks.

Table S1 Results of multivariate linear regression of the relationship between total Isoprene-derived SOA tracers, liquid water content (LWC) of particle, particle acidity (H^+_{aq}), and sulfate for the data of $PM_{2.5}$ in Gucheng.

Variable	β-coefficient	Standard error	t Value	P value
Intercept	44.391	10.801	4.110	<0.001
LWC	-0.580	0.168	-3.462	0.001
H^+_{aq}	-12.859	36.616	-0.351	0.726
Sulfate	4.795	0.595	8.059	<0.001

Table S2 Day-night comparison of the concentrations of total determined isoprene-derived SOA tracers (SOA_i), levoglucosan and sulfate during June 10-18 and June 19-25

	June 10-18		June 19-25	
	Daytime	Nighttime	Daytime	Nighttime
SOA_i (ng m^{-3})	117±86	150±115	87±43	85±53
Levoglucosan (ng m^{-3})	268±347	250±182	137±205	218±208
Sulfate (μg m^{-3})	21±12	26±16	17±15	12±11

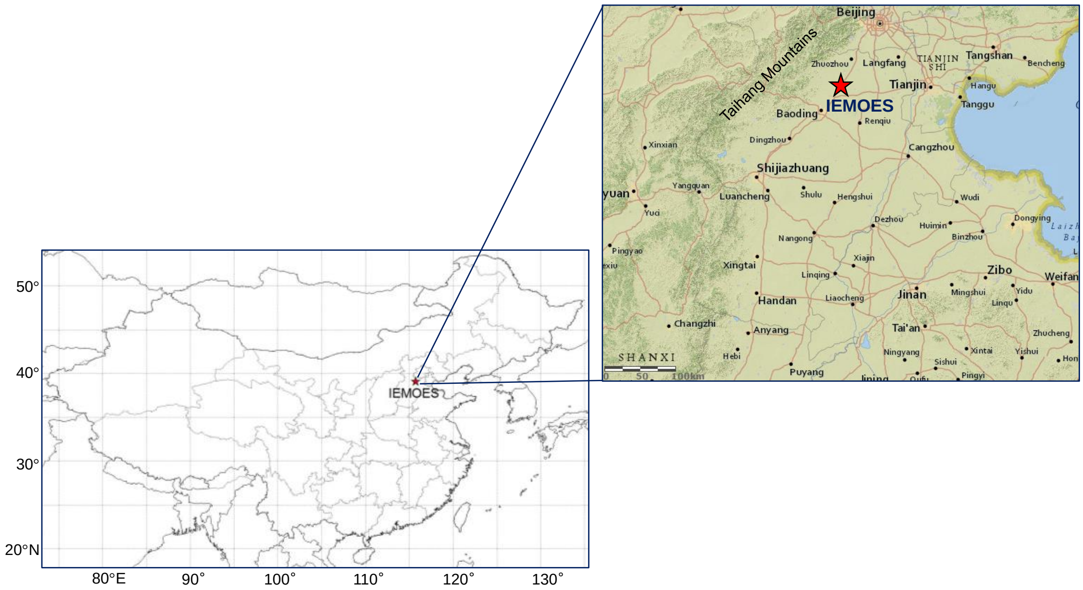

Figure S1. Location of the sampling site (Red star), the Integrated Ecological-Meteorological Observation and Experiment Station (IEMOES) of Chinese Academy of Meteorological Sciences, at Gucheng, Hebei province in northern China. Maps obtained from https://www.arcgis.com/home/webmap/viewer.html and combined by Microsoft Office 2016 – PowerPoint.

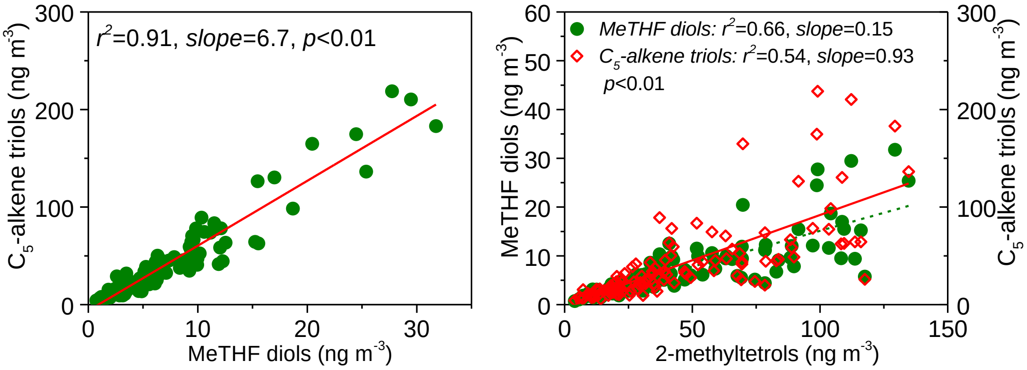

Figure S2. Relationships of IEPOX-derived SOA_i tracers: (a) MeTHF diols and C_5-alkene triols; (b) MeTHF diols and C_5-alkene triols with 2-methyltetrols.

Reference:

Chow, J. C., Watson, J. G., Chen, L. -W. A., Arnott, W. P., Moosmuller, H., Fung, K. K., 2004. Equivalence of elemental carbon by thermal/optical reflectance and transmittance with different temperature protocols. Environmental Science and Technology 38, 4414–4422.

Chow, J.C., Watson, J.G., Chen, L.-W.A., Chang, M.C.O., Robinson, N.F., Trimble, D., Kohl, S., 2007. The IMPROVE_A temperature protocol for thermal/optical carbon analysis: maintaining consistency with a long-term database. Journal of the Air and Waste Management Association 57, 1014–1023.
